# A short note on bioglass in Periodontics

**DOI:** 10.6026/97320630019341

**Published:** 2023-03-31

**Authors:** Vijayalakshmi R, Sruthi Srinivasan, Ambalavanan N

**Affiliations:** 1 Department of Periodontology, Faculty of Dentistry, Meenakshi Ammal Dental College & Hospital, Maduravoyal, Chennai - 600095

**Keywords:** Bone grafts, bioglass, intrabony defect, regeneration, graft associated

## Abstract

Bone augmentation grafts may act as space-maintaining devices to allow coronal migration of periodontal progenitor cells. The ideal bone replacement graft should be able to trigger osteogenesis, cementogenesis and formation of a functional periodontal ligament. It has been theorized that bioactive glass, which is a ceramic has bioactive properties that guide and promote osteogenesis allowing rapid formation of bone. Bioactive glass consists of sodium and calcium salts, phosphates and silicon dioxide for dental applications. When this material comes into contact with tissue fluids, the surface of the particles becomes coated with hydroxy carbonate apatite, incorporates organic ground proteins such as chondroitin sulfate and glycosaminoglycans and attracts osteoblasts that rapidly form bone.

## Background:

Periodontal regeneration is defined as the reproduction/reconstitution of a lost / injured part so that form and function of lost structures are restored (AAP 1992). Bone augmentation grafts may act as space-maintaining devices to allow coronal migration of periodontal progenitor cells. The ideal bone replacement graft should be able to trigger osteogenesis, cementogenesis and formation of a functional periodontal ligament. It has been theorized that bioactive glass, which is a ceramic has bioactive properties that guide and promote osteogenesis allowing rapid formation of bone. Bioactive glass consists of sodium and calcium salts, phosphates and silicon dioxide for dental applications. When this material comes into contact with tissue fluids, the surface of the particles becomes coated with hydroxycarbonate apatite, incorporates organic ground proteins such as chondroitin sulfate and glycosaminoglycans and attracts osteoblasts that rapidly form bone [1].

## Periodontal regeneration:

Periodontal regeneration means healing after periodontal surgery those results in the formation of new attachment apparatus, consisting of cementum, periodontal ligament and alveolar bone. Melcher in 1976 pointed out that the regeneration of the periodontal ligament is the key to new attachment because "it provides continuity between the alveolar bone and the cementum and also because it contains cells that can synthesize and remodel the three connective tissues of the alveolar part of the periodontium." [2] The final outcome of periodontal pocket healing depends on the sequence of events during the healing stages [3]. Only when cells from periodontal ligament proliferate coronally is there new formation of cementum and periodontal ligament [3].

## Events occurring during regeneration of periodontal tissues:

For periodontal regeneration to occur various problems have to be overcome. The first is to alter the periodontitis affected root surface to make it a hospitable substrate to support and encourage migration, attachment, proliferation and proper phenotypic expression of periodontal connective tissue progenitor cells. Mechanical and chemical means have been used to promote favorable root surface characteristics. The next is epithelial exclusion, which is done to prevent the formation of long junctional epithelium, which is formed by proliferation of epithelium apically on the tooth aspect of the flap and becomes attached to the tooth [4]. Bone augmentation grafts have been successfully used to increase the height of periodontal attachment apparatus. Currently, the most often used regenerative techniques involve:

[1] Use of bone-inductive graft materials

[2] Guided cell repopulation using barrier membranes.

[3] Coronally positioned flap procedures in which the flap margin is secured an appreciable distance from healing site.

Indications for periodontal regenerative therapy

[1] To obtain root coverage in order to improve esthetics and reduce root sensitivity.

[2] Furcation-involved teeth

[3] "Hopeless" teeth with deep vertical defects, increased tooth mobility or through and through furcations can be successfully treated with regenerative periodontal therapy [5].

## Osseous defects and classification:

Loss of alveolar bone is one of the characteristic signs of periodontal disease and is generally considered to represent the anatomic sequela to the apical spread of periodontitis. A clear understanding of the topography of bony defects associated with periodontal destruction is essential for diagnosis, prognosis and management. Classifications are generally based upon specific morphological criteria and are aimed at guiding clinicians with their diagnosis, treatment and prognosis [6].

## History of bone grafts:

Bone grafting has been used for almost 100 years in attempts to stimulate healing of bony defects [7]. The first attempt to rebuild bone loss by periodontal disease through bone grafts was reported by Hegedus in 1923 [8]. He initially used bone from the alveolar process, but later he transplanted bone from the tibia over the alveolar process to areas that had been reduced in height by periodontal disease. Nabers and O'Leary revived the method in 1965 and numerous efforts have been made since that time to define its indications and technique. Materials such as plaster of Paris [9], heterogeneous bone powder [10] and other bone preparations [11] were also tried for implantation into intrabony periodontal defects during the 1930's. Since Schallhorn introduced the use of hematopoietic marrow for grafting into Periodontics in 1967great interest in this modality of treatment has been evident [12].

## Bio-active glass [bio-glass]:

Bio-glass is composed of SiO2, Na2 O, P2 O5 and are resorbable or not resorbable depending on the relative proportion of these components. When bio-glasses are exposed to tissue fluids, a double layer of silica gel and calcium phosphate is formed on their surface. Through this layer the material promotes absorption and concentration of proteins used by osteoblasts to form extracellular bone matrix which theoretically may promote bone formation [13]. All bio-active glass produces a strong interface bond with bone. Most of these have a flexural strength, strain to fracture and fracture toughness less than bone. Elastic modulus is greater than both cortical and cancellous bone. This would lead to excessive stress shielding of bone and produce fracture of bone distal and proximal to the implant. So, their use with stress-bearing implants is limited and is restricted to coating metal implants in non-load bearing areas or areas subject to compressive forces. They are used for treatment of periodontal intrabony defects [13] primarily because of their high bioactivity.

## Use of bioactive glasses in the treatment of periodontal intrabony defects:

Studies have found out that the use of bioactive glass allowed the regeneration of a normal periodontium and that it was effective in retarding epithelial down growth than other materials like hydroxyapatite and tricalcium phosphate. The two properties of bioglass that appeared to contribute to these favorable results seem to be first, the increased rate of reaction in vivo that it possesses in comparison to the other materials as a result of its release of silicon and secondly, that it appeared to bond with connective tissue collagen. Because of its high bioactivity, the reaction layers appear to form within minutes of its implantation and the osteogenic cells freed by the surgery can rapidly colonize the particles. This process supplements the bone, which grows by osteoconduction from the alveolus, and these two processes combined have been termed osteoinduction [13]. This results in more rapid filling of the defects than that occurs with other less active materials, such as hydroxyapatite. This may also result from a more rapid accumulation of bone morphogenic proteins and other growth factors on the surface of bio-active particles. Bioglass particulate has also been used to stimulate bone formation in extraction sockets, and to thus maintain the alveolar ridge height [13].

## Healing after placement of bio-active glass:

Immediately after implantation, fibroblasts lay down collagen above the level of the particulate, and this collagen appeared to attach to the superficial particles, immobilizing them in the soft tissue and restoring the transeptal connections of the periodontium. This appeared to prevent epithelial down growth, which only occurs to the point at which it meets adherent collagen fibres overlying the restoring bone. Beneath this layer, the particles induced a production of bone and cementum, and by 9 months the particles were seen within the repairing bone and cementum. A normal periodontal ligament was seen between these tissues [13].

## Future research and trends:

An organic bovine mineral, natural and converted corals, and other materials appear to be suitable as a carrier vehicle for rhBMP2 [14] or other biologic modulators [15], which will increase their usefulness. With source limitations for autogenous bone and patient concerns regarding allogenic bone, the role of bone substitutes will probably increase. The ability to engineer and develop effective, safer, user-friendly synthetic bone replacement graft materials will improve. Bone substitutes are finding increasing use in conjunction with guided tissue barriers to try to improve results with a combined technique. Growing interest in periodontal and other bone regeneration will encourage the development of improved materials.

## Discussion:

The biologic rationale behind the use of bone grafts or alloplastic materials in periodontal regenerative surgery is the assumption that these materials

[1] Contain bone-forming cells [osteogenesis]

[2] Serve as a scaffold for bone formation [osteoconduction]

[3] That matrix of grafting material contains bone inductive substances [osteoinduction]

The various criteria for periodontal regeneration are epithelial exclusion, wound stabilization and use of mechanical and chemical means to promote favorable root surface characteristics. Bone augmentation grafts have been successfully used to increase the height of periodontal attachment apparatus. Regenerative therapy with bone replacement grafts did not gain acceptance as predictable therapy until the 1980's. Controversy still remains as to whether new [attachment] at a coronal level is achieved or whether primarily a long junctional epithelium [repair] occurs as with flap debridement surgery alone.

Among the various methods used for assessing periodontal regeneration, histology remains the ultimate standard. Although routine clinical assessment such as probing or simple measurements from radiographs will be adequate to assess relatively large amounts of regeneration in practice, newer methods such as digital radiography, provide the higher precision needed to detect small differences between different treatment modalities. The two important properties of bioactive glass are first, the increased rate of reaction in vivo that it possesses in comparison to other materials as a result of its release of silicon and secondly, that it appeared to bond with connective tissue collagen.

The shelf life of Perioglas was found to be 3 years and it was resorbable. The mode of breakdown was through leaching and dissolution. Zamet et al. [16] found out that the use of PerioGlas resulted in greater amount of bone fill, with improvements in probing depths and probing attachment levels. However, subsequent human histology has failed to confirm significant new bone or cementum formation following the use of PerioGlas in human intrabony periodontal defects. The shelf life of Biogran was found to be 5 years and it gets resorbed in 6 months if site is loaded. The mode of breakdown is through osteoclasts. Felipe MEM et al. in 2009 conducted a study in dogs in which he analyzed the potential of bioactive glass particles of varying sizes to affect bone formation in periodontal defects and concluded that the use of bioactive glass initiated mineralized bone formation [17]. Surajit Mistry et al. in 2012 [18] compared the efficacy of bioactive glass (BG), hydroxyapatite (HA), and BG-HA composite bone graft particles in the treatment of human infra-bony periodontal defects clinically and radiographically and showed that BG and BG-HA synthetic bone graft implanted sites showed significant bone fill compared to HA and open flap debridement alone for the reconstruction of infrabony defects.

A randomized controlled trial done by El-Haddad et al. in 2014 compared the efficacy of bioactive glass (BG) grafting material versus autogenous bone grafting in the treatment of Grade II furcation involvement. The study concluded that BG and autogenic bone graft had better regenerative attachment gain in the treatment of grade II furcation when compared with open mouth debridement [19]. A meta - analysis study done by Sohrabi et al. in 2012 on the efficacy and effectiveness of B-G materials in regenerative periodontal therapy concluded that the use of BGA in the treatment of intrabony defects showed a significant improvement in probing depth and clinical attachment level [20].

Stavropoulos et al. in 2012 histological evaluated the healing Trans alveolar maxillary sinus augmentation with bioglass and autogenous bone and concluded that the Sinus augmentation with a bioglass and autogenous bone composite is compatible with bone formation [21]. Koller G et al. in 2007 studied the Surface modification caused by bioactive glasses in titanium implants using air abrasion technologies and concluded that the use of BG as a coating material for dental implants produced better outcomes in terms of adherence to the metal surface of implant and bone regeneration [22]. A study done by Talreja et al. in 2013 using collagen as guided bone regeneration barrier in combination with bioactive glass as bone grafting material showed complete resolution of the osseous defect 6 month postoperative, thus concluding that collagen and BGA combination can be used in the treatment of implants undergoing early failure [23]. Studies are presently being conducted to determine if regeneration of periodontal ligament occurs with the use of Biogran; to date, there is no evidence that this occurs in humans.

## Conclusion:

The ultimate aim of periodontal therapy is to achieve periodontal regeneration. The value of using bone grafts or alloplastic materials for regeneration has mainly been examined in case reports, while histologic evidence of new attachment and controlled clinical studies is limited. Bioglass is preferred for its high bioactivity, because of which the reaction layers appear to form within minutes of its implantation and the osteogenic cells freed by the surgery rapidly colonize the particles. Use of bioactive results in more rapid filling of the defects, which may result from more rapid accumulation of bone morphogenic proteins and other growth factors on the surface of bioactive glass. The prevention of epithelial down growth is probably the main advantage of using bioactive glass. At present, bone substitutes are finding increasing use in conjunction with guided tissue barriers. Growing interest periodontal and other bone regeneration will encourage the development of improved materials.
